# A Longitudinal Study on the Effects of Parental Monitoring on Adolescent Antisocial Behaviors: The Moderating Role of Adolescent Empathy

**DOI:** 10.3389/fpsyg.2016.01726

**Published:** 2016-11-03

**Authors:** Elisabetta Crocetti, Jolien Van der Graaff, Silvia Moscatelli, Loes Keijsers, Hans M. Koot, Monica Rubini, Wim Meeus, Susan Branje

**Affiliations:** ^1^Research Centre Adolescent Development, Utrecht UniversityUtrecht, Netherlands; ^2^Department of Psychology, University of BolognaBologna, Italy; ^3^Department of Developmental Psychology, Tilburg UniversityTilburg, Netherlands; ^4^Department of Clinical Developmental Psychology, EMGO Institute for Health and Care Research, Vrije Universiteit AmsterdamAmsterdam, Netherlands

**Keywords:** parental solicitation, parental control, adolescent antisocial behaviors, empathy, longitudinal, moderation

## Abstract

In adolescence, youth antisocial behaviors reach a peak. Parents can use different strategies, such as parental solicitation and control, to monitor their children’s activities and try to prevent or reduce their antisocial behaviors. However, it is still unclear if, and for which adolescents, these parental monitoring behaviors are effective. The aim of this study was to examine if the impact of parental solicitation and control on adolescent antisocial behaviors depends on adolescent empathy. In order to comprehensively address this aim, we tested the moderating effects of multiple dimensions (affective and cognitive) of both trait and state empathy. Participants were 379 Dutch adolescents (55.9% males) involved in a longitudinal study with their fathers and mothers. At T1 (conducted when adolescents were 17-year-old) adolescents filled self-report measures of antisocial behaviors and trait empathy during one home visit, while their state empathy was rated during a laboratory session. Furthermore, parents reported their own monitoring behaviors. At T2 (conducted 1 year later, when adolescents were 18-year-old), adolescents reported again on their antisocial behaviors. Moderation analyses indicated that both affective and cognitive state empathy moderated the effects of parental solicitation on adolescent antisocial behaviors. Results highlighted that solicitation had unfavorable effects on antisocial behaviors in adolescents with high empathy whereas the opposite effect was found for adolescents with low empathy. In contrast, neither state nor trait empathy moderated the effects of control on adolescent antisocial behaviors. Theoretical and practical implications of these findings are discussed.

## Introduction

In adolescence, youth antisocial behaviors reach a peak (e.g., Farrington, 1986; Emler and Reicher, 1995; Meeus et al., 2016). Although for most individuals, these behaviors are transitory, limited to the adolescent phase, and sharply declining in young adulthood, a limited portion of adolescents shows life-course persistent antisocial behaviors (Moffitt, 1993). These behaviors are highly costly for the society and for the individual (Racz and McMahon, 2011), hampering youth positive development (e.g., Crocetti et al., 2013). Thus, parents, educators, practitioners, and scholars are interested in knowing how to lessen these behaviors and prevent them from becoming life-persistent.

For a long time, the view that parents could prevent adolescents’ antisocial behaviors, by providing adolescents with guidance and control, dominated the field (Loeber and Dishion, 1983; Patterson and Stouthamer-Loeber, 1984). The last decades have witnessed a shift to a more comprehensive and dynamic view of parent–adolescent interactions (e.g., Lollis and Kuczynski, 1997; Kerr et al., 2003), focusing on how adolescents’ behaviors can predict changes in parenting (e.g., Kerr and Stattin, 2003; Branje et al., 2008; Sameroff, 2009; Keijsers et al., 2010; Kerr et al., 2012; Crocetti et al., 2016) and how adolescents’ characteristics can explain why and when parental behaviors might be more or less beneficial (Belsky, 2005; Boyce and Ellis, 2005; Ellis et al., 2011). Improving our understanding of this latter aspect has important theoretical and practical implications, as it informs us about which parental practices might be useful for which adolescents.

In line with these considerations, in this study we sought to examine whether adolescent empathy moderates the effects of strategies that parents can use to monitor their children (Kerr and Stattin, 2000) on adolescent antisocial behaviors. Empathy is a fundamental social skill, representing the ability to understand and to share the emotional state of another person, and it is considered a main pillar of morality (e.g., Miller and Eisenberg, 1988; Hoffman, 2000). We hypothesized that adolescent empathy could moderate the effects of parental monitoring on adolescent antisocial behaviors, and tested this using a multi-informant multi-method longitudinal design.

### Parental Monitoring and Adolescent Antisocial Behaviors

During adolescence, parents use different strategies to be informed about their children’s activities, friends, and whereabouts (Kerr and Stattin, 2000). Specifically, two practices parents can use to monitor their children are *parental solicitation*, referring to the extent to which parents ask children for information, and *parental control*, referring to the extent to which parents impose rules and restrictions on adolescents’ behaviors, thereby limiting their freedom of doing activities without informing their parents (Stattin and Kerr, 2000). Thus, parents can try to receive information from their children through different behaviors.

Effects of parental solicitation and control on adolescents’ antisocial behaviors are not clear (reviews: Racz and McMahon, 2011; Keijsers, 2015). In fact, some longitudinal studies did not find associations between solicitation and control and later problem behaviors (e.g., Keijsers et al., 2010; Kerr et al., 2010; Stavrinides et al., 2010), but others found that solicitation (e.g., Kiesner et al., 2009; Willoughby and Hamza, 2011) and control (e.g., Willoughby and Hamza, 2011; Wang et al., 2013) predicted higher levels of problem behaviors. Thus, the pattern of associations between monitoring practices and adolescent antisocial behaviors is rather inconsistent.

These inconsistent results might be explained by the fact that these parental behaviors are effective for some adolescents, but not for others (Laird et al., 2010). This is line with theories on differential susceptibility (Belsky, 2005; Boyce and Ellis, 2005; Ellis et al., 2011), according to which child characteristics can make children more or less likely to benefit from certain contextual influences, in this case parental monitoring. In particular, children’s empathy can explain why some adolescents perceive certain parental behaviors as legitimate whereas others may view these behaviors as intrusive and inappropriate (e.g., Keijsers and Laird, 2014). Therefore, we sought to shed light on this by testing whether adolescent empathy would moderate the effects of parental control and solicitation on adolescent antisocial behaviors.

### The Potential Moderating Role of Empathy

Empathy is a multi-dimensional construct, involving affective and cognitive processes that both concern responses of one individual to the experiences of another (e.g., Davis, 1996; Batson, 2009; Decety and Cowell, 2014). Affective empathy refers to the vicarious experience of emotions consistent with those of the observed person (Cohen and Strayer, 1996; Hoffman, 2000) and often results in *empathic concern*, which involves feelings of sorrow or concern for another (Eisenberg, 2000). Cognitive empathy, or *perspective taking*, can be defined as the ability to understand others’ emotions (Davis, 1983). Furthermore, in addition to the distinction between affective and cognitive empathy, it is relevant to differentiate between *trait empathy*, referring to an individual’s general tendency to empathize with others, and *state empathy*, referring to the transient empathic reaction elicited in specific situations. Thus, empathy is a multi-faceted phenomenon.

Previous research revealed modest interrelations between these different dimensions of empathy (Van der Graaff et al., 2016) and suggested that different empathy dimensions can carry diverse implications for adolescents’ social functioning, including antisocial behaviors (van Noorden et al., 2015). For instance, previous correlational (e.g., Jolliffe and Farrington, 2011), longitudinal (e.g., Batanova and Loukas, 2014), and experimental (e.g., Edele et al., 2013) studies indicated that affective empathy might be more relevant than cognitive empathy in explaining youth behaviors. Also, associations between empathy and aggression have been found to differ depending on whether *trait* or *state* empathy was assessed (see Miller and Eisenberg, 1988). Thus, it is important to adopt a multi-dimensional approach in the study of empathy (Batson, 2009; Decety and Cowell, 2014) and of its effects for adolescent psychosocial development.

In particular, empathy makes adolescents more attuned to relationships (Hoffman, 2000). Thus, adolescents could respond differently to parental monitoring according to how they interpret these behaviors. So, adolescent empathy could be a key factor that explains why some adolescents consider parental monitoring behaviors as non-legitimate and intrusive, whereas others consider them legitimate interference in their lives (e.g., Smetana et al., 2005).

In line with this reasoning, in this study we examined whether different dimensions of empathy could moderate associations between parental monitoring and adolescent antisocial behaviors. Some empirical studies have shown that adolescents with different levels of empathy can indeed benefit differently from the relationships with their parents. For instance, Van der Graaff et al. (2012) found that adolescent trait empathic concern moderated the associations between parental support and antisocial behavior: highly empathic 14- to 15-year-old adolescents appeared to be more susceptible to the beneficial effects of parental support than their peers with lower levels of empathy. High empathic adolescents, however, can also be more sensitive to conflictual and problematic family relationships (Van Lissa, 2016). In line with this, another study found that maternal criticism was positively related to child conduct problems only for children high in empathy (Miller et al., 2014). Thus, extant evidence suggests that empathy can amplify adolescents’ responses to the quality of parent–adolescent relationships, either for better or for worse.

Building upon this background, competing hypotheses can be advanced. In fact, drawing from the basic principle that empathy makes adolescents more attuned to relationships (Hoffman, 2000) we can hypothesize that adolescents with higher empathy (a) might be better able to see parents’ good intentions in solicitation and control; or (b) might be more sensitive to the potentially intrusive aspects of solicitation and control in a period of adolescence in which both have to decline to match youth autonomy needs (Racz and McMahon, 2011). In line with the first hypothesis, solicitation and control would be negatively related to antisocial behaviors in high empathic adolescents. In contrast, in line with the second hypothesis, solicitation and control would be positively related to antisocial behaviors in high empathic adolescents. This second hypothesis implies that high empathic adolescents could become oppositional and display antisocial behaviors in response to parental behaviors that might be perceived as intrusive. We sought to test both competing hypotheses to clarify if and for which adolescents parental monitoring behaviors are more or less beneficial.

In testing these hypotheses, we focused on the developmental period of late adolescence (from 17 to 18 years old), as in this period, parental solicitation and especially control are expected to decline (Keijsers et al., 2009), in response to adolescents’ increasing need for autonomy. Thus, in late adolescence children might perceive parental solicitation and control as less appropriate than in prior early and middle adolescent phases. So, the focus on this period offers the best context for testing our competing hypotheses, providing the opportunity to capture adolescents’ more nuanced ways of interpreting parental monitoring behaviors on the basis of their different levels of empathy.

### The Present Study

**FIGURE 1:**
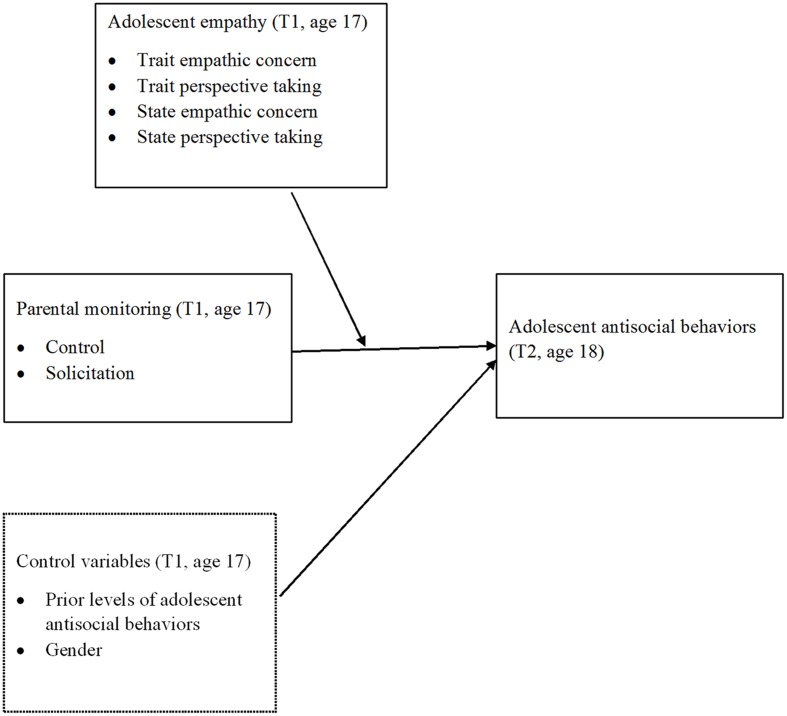
**The conceptual moderated model**.

In synthesis, the aim of the present longitudinal multi-method and multi-informant study was to examine if the effects of parental monitoring on adolescent antisocial behaviors would be moderated by adolescent empathy. In line with recent developments of the empathy literature (e.g., Decety and Cowell, 2014; Van der Graaff et al., 2016), we tested for the moderating effects of trait and state dimensions of empathy considering both empathic concern and perspective taking. More specifically, as schematized in **Figure 1**, we investigated whether the effects of parental control and solicitation reported by fathers and mothers at T1 (when adolescents were 17-year-old) were differently related to relative changes (controlled for prior levels) in adolescent antisocial behaviors at T2 (1 year later, when adolescents were 18-year-old), for different levels of adolescent empathy at T1.

## Materials and Methods

### Participants and Procedure

Data were drawn from the ongoing longitudinal RADAR-young study (Research on Adolescent Development and Relationships—younger cohort), a population-based prospective cohort study conducted in Netherlands. Participants for the current study were 379 target adolescents (55.9% males; *M*_age_ = 17, *SD*_age_ = 0.44), their fathers (*M*_age_ = 51.01, *SD*_age_ = 5.49), and mothers (*M*_age_ = 48.37, *SD*_age_ = 4.47). All participating adolescents were attending secondary schools. Most adolescents were native Dutch (95.8%), lived with both parents (78.4%), and came from families classified as medium or high socioeconomic status (91.8%).

A check on missing data indicated that 90% of adolescents and 80% of parents filled more than 80% of study items. Little’s (1988) Missing Completely at Random (MCAR) test yielded non-significant results for both adolescents’, χ^2^ (15) = 17.224, *p* = 0.306 (χ^2^/df = 1.15) and parents’ data, χ^2^ (7) = 6.364, *p* = 0.498 (χ^2^/df = 0.91). This indicates that data were missing completely at random and, thus, missing data could be safely estimated in SPSS with the EM algorithm.

### Procedure

The RADAR study has been approved by the Medical Ethical Committee of Utrecht University Medical Centre (Netherlands). Data for the current study were collected during two home visits (conducted when adolescents were 17- and 18-year-old) and one laboratory session (conducted when adolescents were 17-year-old). In the first home visit, adolescents rated their own antisocial behaviors and trait empathy and both parents reported about their parental monitoring practices. In the laboratory session, state empathy was assessed using an empathy task. In the second home visit, adolescents rated again their antisocial behaviors. Before the start of the study, adolescents and their parents received written information about the study and they provided their written informed consent in accordance with the Declaration of Helsinki, separately for participation in the home visit and the laboratory session. Adolescents received 30 Euros for participation in each home visit, and 50 Euros for participation in the laboratory session.

### Measures

#### Adolescent Antisocial Behaviors

Adolescents reported their antisocial behaviors both at T1 and T2 filling out the Dutch version (Verhulst et al., 1997) of the antisocial behaviors subscale (30 items) of the Youth Self-Report (Achenbach, 1991). Sample items include “I destroy things that belong to others” and “I steal at home.” All items were scored on a 3-point scale: 0 (never), 1 (sometimes), and 2 (often). In this study, Cronbach’s alpha was 0.88, both at T1 and at T2.

#### Parental Monitoring Behaviors

Both fathers and mothers reported their own control and solicitation at T1, completing the Dutch version (cf. Hawk et al., 2008) of the monitoring scales developed by Kerr and Stattin (Kerr and Stattin, 2000; Stattin and Kerr, 2000). Sample items include: “Does your child have to ask you before he or she can make plans to do something with friends on a Saturday night?” (control, five items); and “During the past month, how often have you started a conversation with your child about his or her free time?” (solicitation, three items). All items were scored on a 5-point scale, ranging from 1 (never) to 5 (always). Maternal and paternal reports were combined to obtain overall parents’ control and solicitation. In this study, Cronbach’s alphas were 0.87 for control and 0.66^1^ for solicitation, respectively.

#### Adolescent Trait Empathy

Adolescents reported on their own trait empathy at T1, using two subscales (empathic concern and perspective taking) of the Dutch version (Hawk et al., 2013) of the Interpersonal Reactivity Index (IRI; Davis, 1980, 1983). Sample items include: “I often have tender, concerned feelings for people less fortunate than me” (empathic concern, seven items) and “I try to look at everybody’s side of a disagreement before I make a decision” (perspective taking, seven items). All items were scored on a 5-point scale, ranging from 0 (does not describe me at all) to 4 (describes me very well). In this study, Cronbach’s alphas were 0.71 for empathic concern and 0.78 for perspective taking.

#### Adolescent State Empathy

When adolescents were 17 years old (T1), they visited the university to participate in an individual test session during which empathic responses to emotional film clips were assessed. The session took place in a testing room equipped with a personal computer to present the stimulus material and to record participants’ self-reported responses to the film clips. An adjacent observation room with a one-way mirror was equipped with a personal computer for control of the experiment. Following a written protocol, a trained female experimenter demonstrated the computerized empathy task and gave instructions for completing ratings after each film clip (see below). The experimenter then dimmed the light and left the testing room after which the participant watched the film clips and completed the questions.

During the test session, participants were exposed to empathy-inducing film clips, assembled from Dutch documentary films, in which different emotions were portrayed (De Wied et al., 2012) to assess state empathy. The current study uses data from the two film clips that represented sadness (i.e., a girl sent to a boarding school, and a boy who is rejected to join a select soccer team). The film clips (length, respectively, 153 and 156 s) each started with a voice-over sketching the situation and ended with a target scene in which the central figure portrayed intense facial and vocal expressions of sadness.

A computerized procedure, adapted from Strayer’s (1993) Empathy Continuum, was used to assess adolescents’ affective and cognitive responses to the emotional film clips (De Wied et al., 2005). After each film clip questions were asked about the quality and intensity of observed and experienced emotions, on which scores on state empathic concern and state perspective taking were based. First participants were asked to identify the emotion expressed by the protagonist by marking one or more pictograms portraying: (1) fear, (2) anger, (3) happiness, (4) sadness, (5) surprise, or (6) neutral/no emotion. For both film clips almost all participants in the current study (99.2%) correctly identified “sadness” as the prominent emotion. Thus, individual differences in empathic responses to the film clips cannot be attributed to differences in emotion recognition.

##### State empathic concern

Participants were asked whether they felt sorry (yes/no) for the protagonist. If they indicated “yes,” they were asked to indicate the intensity of this feeling on a scale ranging from 1 (a little) to 4 (very much). In the current study, state empathic concern responses refer to the intensity of the feelings of concern, scored on a 5-point scale (0 = no empathic concern, 4 = very much). Scores were averaged across the two film clips. 88.4% of the participants received a score higher than 0 on state empathic concern.

##### State perspective taking

If participants had indicated that they felt sorry for the protagonist, they were asked to explain aloud why they sympathized. The answers were recorded and were coded by two trained coders on an 8-point scale, in accordance to Strayer’s (1993) Empathy Continuum. Respondents received a score 0 if they did not correctly identify the target emotion experienced by the protagonist. If participants identified the emotion correctly, but reported no empathic concern (answer “no” to the question whether they felt sorry for the protagonist) they received score 1. The presence of a sympathetic response, and a correct identification of the protagonist’s emotion were necessary to get a score level 2 or higher on the state perspective taking scale: 2 = no or irrelevant attribution, 3 = attribution based on events only, 4 = minimal mention of the stimulus person in the event, 5 = attribution indicating association to own experience, 6 = attribution indicating responsiveness to character’s internal state or general life situation, 7 = attribution indicating explicit role taking. The responses to the two film clips were coded by (a) a research assistant that, after receiving a specific training, coded all the data, and (b) the second author that coded two subsets of data. Inter-scorer reliabilities were established on the two subsets of data coded by both coders: this was 36% of the data of the first film clip (Cohen’s kappa = 0.88), and 40% of the second film clip (kappa = 0.83). Scores on state perspective taking were then averaged across the two film clips.

## Results

### Preliminary Analyses

Descriptive statistics (means and standard deviations) and bivariate correlations among study variables are reported in **Table 1**. Findings highlighted a significant (*p* < 0.01) decrease in adolescent antisocial behaviors, however, the effect size of this change was very small (Cohen’s *d* = -0.10). Furthermore, results indicated that parental control and solicitation were moderately correlated and they were unrelated to adolescent empathy and antisocial behaviors. Trait empathic concern and perspective taking were strongly interrelated and both of them were negatively associated with adolescent antisocial behaviors. State empathic concern and perspective taking were also highly interrelated but they were unrelated to adolescent antisocial behaviors. Associations between trait and state dimensions of empathy were small. Finally, the correlation between adolescent antisocial behaviors at T1 and T2 was very strong, highlighting high stability.

**Table 1:** Descriptive statistics and bivariate correlations among study variables.

	Response scale	*M* (*SD*)	2	3	4	5	6	7	8
1. Parental control (T1)	1–5	3.27 (0.83)	0.29^∗∗∗^	-0.00	-0.05	-0.04	-0.05	0.07	0.09
2. Parental solicitation (T1)	1–5	3.56 (0.47)	1	-0.00	-0.09	0.03	0.01	-0.07	-0.05
3. Adolescent trait empathic concern (T1)	0–4	2.45 (0.56)		1	0.52^∗∗∗^	0.19^∗∗∗^	0.14^∗∗^	-0.28^∗∗∗^	-0.23^∗∗∗^
4. Adolescent trait perspective taking (T1)	0–4	2.22 (0.61)			1	0.07	0.14^∗∗^	-0.39^∗∗∗^	-0.22^∗∗∗^
5. Adolescent state empathic concern (T1)	0–4	2.19 (0.83)				1	0.59^∗∗∗^	-0.05	0.02
6. Adolescent state perspective taking (T1)	1–7	4.53 (1.45)					1	-0.07	-0.08
7. Adolescent antisocial behaviors (T1)	0–2	0.34 (0.25)						1	0.73^∗∗∗^
8. Adolescent antisocial behaviors (T2)	0–2	0.32 (0.24)							1


**M*, mean; *SD*, standard deviation. ^∗∗^*p* < 0.01, ^∗∗∗^*p* < 0.001.*

### Moderation Analyses

The aim of this study was to examine whether associations between parental monitoring and adolescent antisocial behaviors were moderated by adolescent empathy. In order to reach this goal, we performed statistical analyses in SPSS by means of the PROCESS macro 2.13.2 (Hayes, 2013). Specifically, we conducted analyses using model 1 of PROCESS, in which it is possible to test whether the effect of one independent variable (parental control or parental solicitation) on one outcome variable (adolescent antisocial behaviors at T2) is moderated by one variable (one dimensions of empathy) controlling for one or more covariates (antisocial behaviors at T1 and sex dummy coded). Both the independent and the moderator variables were mean centered prior to analyses. We used 1,000 bootstrap estimates for the construction of 95% bias-corrected CIs for the effect estimates. Furthermore, we obtained regions of significance with the Johnson–Neyman technique that yields statistical significance transition points within the observed range of the moderator. Finally, to facilitate interpretation of significant moderations we plotted conditional effects (simple slopes) for low (sample mean -1 SD), medium (sample mean), and high (sample mean +1 SD) levels of the moderating variables.

We ran a total of eight regression analyses (one for each combination of one parental monitoring behavior and one dimension of empathy). Overall, findings highlighted no main effects of parental control and solicitation on adolescent antisocial behaviors. Importantly, trait empathy did not have moderating effects whereas state empathy was found to be a significant moderator of the association between parental monitoring and later adolescent antisocial behaviors in 50% of the analyses. These results were controlled for prior levels of antisocial behaviors (that represented a very strong and significant predictor of later antisocial behaviors) and gender (that resulted to be unrelated to later antisocial behaviors).

**FIGURE 2:**
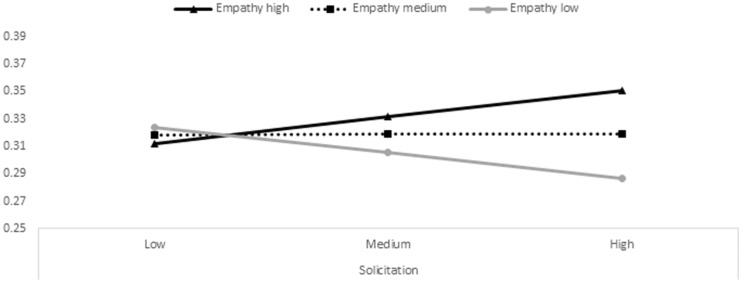
**Association between paternal solicitation at T1 (on the *x*-axis) with adolescent antisocial behaviors at T2 (on the *y*-axis) for low (gray line), medium (dashed line), and high (black line) levels of state empathic concern**.

**FIGURE 3:**
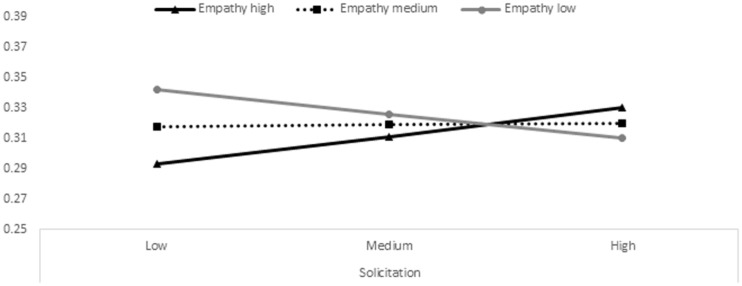
**Association between paternal solicitation at T1 (on the *x*-axis) with adolescent antisocial behaviors at T2 (on the *y*-axis) for low (gray line), medium (dashed line), and high (black line) levels of state perspective taking**.

As reported in **Table 2**, state empathic concern and state perspective taking moderated the association between parental solicitation and adolescent antisocial behaviors. More specifically, moderator value(s) defining Johnson–Neyman significance region(s) highlighted that for high levels of state empathic concern (0.92 standard deviations above mean) and state perspective taking (1.89 standard deviations above mean) parental solicitation became significantly positively related to adolescent antisocial behaviors, whereas for low levels of state empathic concern (1.07 standard deviations below mean) parental solicitation became significantly negatively related to adolescent antisocial behaviors. **Figures 2** and **3** visualize these moderation effects by plotting simple slopes for adolescents with high, medium, and low empathy scores. Thus, these findings indicate that for youth with high state empathic concern and perspective taking, higher levels of parental solicitation were related to higher levels of antisocial behaviors; whereas for adolescents with low state empathic concern, the correlation was the reverse^2^.

**Table 2:** Results of moderation analyses.

	Interaction effect [95% CI]	Moderator value(s) defining Johnson–Neyman significance region(s)
**Association between parental control and adolescent antisocial behaviors moderated by:**		
Trait empathic concern	-0.01 [-0.04, 0.03]	None
Trait perspective taking	0.00 [-0.03, 0.03]	None
State empathic concern	0.02 [-0.00, 0.04]	None
State perspective taking	0.01 [-0.01, 0.02]	None
**Association between parental solicitation and adolescent antisocial behaviors moderated by:**		
Trait empathic concern	0.01 [-0.06, 0.07]	None
Trait perspective taking	-0.03 [-0.09, 0.03]	None
State empathic concern	0.05^∗∗^ [0.01, 0.08]	1.07 SD below the mean; 0.92 SD above the mean
State perspective taking	0.03^∗^ [0.00, 0.05]	1.89 SD above the mean


*^∗^*p* < 0.05, ^∗∗^*p* < 0.01.*

## Discussion

In this study, we shed new light on differential effects of parental monitoring on adolescent antisocial behaviors. We found that both affective (empathic concern) and cognitive (perspective taking) state empathy moderate the effects of parental solicitation on adolescent antisocial behaviors. Results highlighted that solicitation had unfavorable effects on adolescent antisocial behaviors in adolescents with high empathy whereas the opposite effect was found for adolescents with low empathy. In contrast, neither state nor trait empathy moderated the effects of control on adolescent antisocial behaviors. Theoretical and practical implications of these findings are discussed below.

### Differential Associations between Parental Monitoring and Adolescent Antisocial Behaviors: Empathy Moderating Effects

Findings pointed out that parental monitoring behaviors, reported directly by adolescents’ parents, did not have a main effect on their children’s later antisocial behaviors. Thus, in line with prior longitudinal studies (Keijsers et al., 2010; Kerr et al., 2010; Stavrinides et al., 2010), the association between parental solicitation and control and adolescent problems was not straightforward. Importantly, in this study we found that this lack of a significant association might hide a moderation effect, implying that these monitor behaviors might be beneficial for some adolescents and detrimental for others (Laird et al., 2010). Thus, in line with theories on differential susceptibility (Belsky, 2005; Boyce and Ellis, 2005; Ellis et al., 2011), parental influences can be better understood taking also into account adolescents’ characteristics.

We found that the impact of parental solicitation on children’s antisocial behaviors differed for adolescents with high and low state empathy. More specifically, parent-reported parental solicitation was related to a relative increase in adolescent-reported antisocial behaviors 1 year later, controlling for prior levels of antisocial behaviors and gender, in adolescents with high state empathy. This result was found for both affective (empathic concern) and cognitive (perspective taking) state empathy. In contrast, parental solicitation was related to lower relative levels of antisocial behaviors in adolescents with low affective state empathy. These findings suggest a number of considerations.

First, results indicate that adolescents with higher empathic skills were more likely to react aversively to high levels of parental solicitation. Thus, it is more important for parents to attune their monitoring to the developing autonomy needs of adolescents when adolescents have high interpersonal skills and are, therefore, more attentive to the potential intrusive nature of these behaviors. In line with recent development of the empathy literature, this result points to the potential downside of empathy (Righetti et al., 2016; Van Lissa, 2016). In fact, the interpersonal sensitivity associated with high empathy may enable adolescents to experience even milder forms of parental monitoring as intrusive and violating their privacy (Hawk et al., 2008). That we found moderating effects for parental solicitation but not for control could be due to the fact that whereas high parental control is a more explicit and severe behavior that is largely perceived as intrusive by adolescents (Kakihara and Tilton-Weaver, 2009; Kakihara et al., 2010), solicitation is a more nuanced monitoring behavior. Thus, adolescents can experience solicitation differently, as more or less appropriate, according to their interpersonal skills.

Second, we found beneficial effects of parental solicitation for adolescents with low state empathic concern. This is in line with Laird et al.’s (2010) suggestion that parental monitoring might be more effective when needed most. In fact, Laird et al. (2010) found that the effects of parental solicitation (but not control) on adolescent antisocial behaviors were moderated by unsupervised time spent with peers in such a way that adolescents’ perceptions of greater solicitation were more strongly associated with lower levels of antisocial behaviors among adolescents spending more time unsupervised. Akin, in this study we found that parental solicitation is more beneficial for low empathic adolescents that might need a stronger parental presence for regulating their behavior and reduce involvement in antisocial acts.

Third, our findings were different for trait versus state dimensions of empathy. In line with a wide literature (e.g., Jolliffe and Farrington, 2004; de Kemp et al., 2007; Gini et al., 2007; Ang and Goh, 2010; van Noorden et al., 2015), we found significant negative correlations, both concurrently and over time, between trait (but not state) dimensions of empathy and adolescent antisocial behaviors, consistent with previous research in which externalizing behavior appeared more consistently related to self-reported measures of trait empathy than to state empathy measures (see Miller and Eisenberg, 1988). On the other hand, state (but not trait) dimensions of empathy moderated the impact of parental solicitation on antisocial behaviors. When compared to trait empathy self-report measures, our measures of state empathy are less likely to be influenced by social desirability and self-presentational biases (Van der Graaff et al., 2016). Moreover, whereas the trait empathy measures tap into adolescents’ empathic tendency, or their general motivation to respond with empathy, the state empathy measures assess adolescents’ actual empathic responsiveness as evoked by real life emotional situations. Therefore, state empathy may better reflect adolescents’ actual sensitivity and responsiveness to parental solicitation.

Finally, we found a significant moderation for both affective and cognitive dimensions of state empathy. For affective empathy, we found differential significant effects at high and low levels, whereas for cognitive empathy, we detected a significant effect only at high levels. Overall, these results suggest that empathic concern might be slightly more relevant in disentangling differential effects of parental solicitation on adolescent antisocial behaviors than is perspective taking and further points to the importance of studying empathy as a multi-dimensional phenomenon (Batson, 2009; Decety and Cowell, 2014).

### Strengths, Limitations, and Suggestions for Future Research

This study should be considered in light of both its strengths and limitations, from which it is possible to suggest future lines of research. A first strength of this study was its longitudinal design involving both the target adolescents and their parents. This design allowed us to examine if and how empathy moderates the effects of parental monitoring at T1 on levels of adolescent antisocial behaviors at T2 controlling for prior levels of antisocial behaviors (Adachi and Willoughby, 2015). Importantly, since parental monitoring behaviors were reported directly by adolescents’ parents we can exclude reporting biases.

A further strength of this study was the inclusion of different types and dimensions of empathy (Decety and Cowell, 2014). In this way, we could highlight the specific role played by trait and state dimensions, considering both cognitive and affective facets. Since most of these dimensions of empathy are modestly related (Van der Graaff et al., 2016) future studies could further advance this line of research adopting a person-centered approach to distinguish adolescents with distinct empathy *profiles* (e.g., adolescents high in trait but low in state empathy). Doing so, it would be possible to test if and how associations between parental monitoring and adolescent antisocial behaviors are moderated by empathy profiles.

A limitation of this study was the sample composition. In fact, our sample consisted of Dutch families with medium to high socio-economic status. Thus, findings cannot be generalized behind this specific population. Future research is needed to test whether findings of this study can be replicated in different cultural contexts and types of families. In fact, parental monitoring behaviors can be interpreted differently by adolescents according to the extent to which they match cultural values (for a discussion, see Grusec, 2008). For instance, in collectivist Asian countries adolescents are more likely to interpret even high levels of parental control as expressions of warmth and caring (Chao and Tseng, 2002; Kakihara et al., 2010). Thus, it could be the case that the moderating role of empathy described in the current study works differently in collectivistic Asian youth, in which high empathic adolescents might emphasize more than low empathic adolescents the positive intentions behind parents’ solicitation and control, thus benefitting the most from them.

Furthermore, this study involved 17-year-old adolescents (followed until they were 18-year-old). To focus on this developmental period is of clear importance, since parental monitoring behaviors are expected to decline in this period (Keijsers et al., 2009). Therefore, in middle-to-late adolescence these behaviors might be considered as less normative. However, it is also important to further understand if and how the impact of parental monitoring differs for adolescents with high and low empathy also in early-to-middle adolescence (e.g., Laird et al., 2010). In fact, the onset of adolescence might be another particularly sensitive period for unraveling this issue since parents and offspring need to renegotiate their relationship, finding a new balance that is appropriate for adolescents’ increasing need for autonomy (Laursen and Collins, 2009).

## Conclusion

In this prospective longitudinal study, we found that parental solicitation behaviors aimed at eliciting adolescents’ disclosure of information had detrimental effects for adolescents with high state empathy, while they were more positive for those with low empathy. These results have relevant practical implications, suggesting that parents need to become aware that their behaviors might elicit different effects for different children. Thus, they need to modulate their solicitation behaviors, using them with caution with high empathic children that can experience them as a violation of their privacy and a lack of trust. Overall, this study further contributes to the theoretical understanding of the active role played by adolescents’ characteristics in disentangling the dynamic interplay between parent–child relationship and adolescent psychosocial development (Belsky, 2005; Sameroff, 2009; Ellis et al., 2011).

## Author Contributions

EC and SB conceived the current study; EC performed the statistical analyses and wrote the manuscript; all authors participated in the interpretation of the results and in the drafting of the article; SB, HK, and WM are the principal investigators of the RADAR project and are responsible for the data collection; JV collected the laboratory data. All authors read and approved the final manuscript.

## Conflict of Interest Statement

The authors declare that the research was conducted in the absence of any commercial or financial relationships that could be construed as a potential conflict of interest.

## Footnotes

1 The value of the Cronbach’s alpha for solicitation is similar to those reported in prior studies (e.g., Keijsers et al., 2010). Although this value is moderately low, it can still be considered acceptable. The consequences of having a moderately low alpha would be to underestimate the effect under investigation (Hunter and Schmidt, 2004) and yield more conservative results (i.e., to reduce the likelihood of finding results that are statistically significant). In this study, as further reported in the Section “Results,” we found that half of the moderation tests including solicitation resulted to be statistically significant, although the value of the alpha was moderately low. Thus, this further supports the robustness of study findings.

2 In line with relevant suggestions provided by an anonymous reviewer, we checked whether our findings could be influenced by general parenting climate (using support as reported by parents as a proxy of it). First, we have re-run all the analyses controlling for general parenting climate. Support was used as a covariate of both empathy and adolescent antisocial behaviors. In all these new analyses, we found that the effect of support was not significant and any of the results reported in the Section “Results” changed after controlling for support. Additionally, in a second set of ancillary analyses, we checked whether, as suggested by the reviewer, parental support could a moderator of the association between parental monitoring behaviors and adolescent antisocial behaviors. Results indicated that in this study support was not a significant moderator of the association between parental solicitation and control and adolescent antisocial behaviors.
